# Shearing of the CENP-A dimerization interface mediates plasticity in the octameric centromeric nucleosome

**DOI:** 10.1038/srep17038

**Published:** 2015-11-25

**Authors:** David Winogradoff, Haiqing Zhao, Yamini Dalal, Garegin A. Papoian

**Affiliations:** 1 Chemical Physics Program, University of Maryland, College Park, MD 20742, USA; 2 Biophysics Program, University of Maryland, College Park, MD 20742, USA; 3 Laboratory of Receptor Biology and Gene Expression, National Cancer Institute, National Institutes of Health, Bethesda, MD, USA; 4 Department of Chemistry and Biochemistry, University of Maryland, College Park, MD 20742, USA

## Abstract

The centromeric nucleosome is a key epigenetic determinant of centromere identity and function. Consequently, deciphering how CENP-A containing nucleosomes contribute structurally to centromere function is a fundamental question in chromosome biology. Here, we performed microsecond timescale all-atom molecular dynamics (MD) simulations of CENP-A and H3 nucleosomes, and report that the octameric CENP-A core particles and nucleosomes display different dynamics from their canonical H3-containing counterparts. The most significant motion observed is within key interactions at the heart of the CENP-A octameric core, wherein shearing of contacts within the CENP-A:CENP-A’ dimerization interface results in a weaker four helix bundle, and an extrusion of 10–30 bp of DNA near the pseudo-dyad. Coupled to other local and global fluctuations, the CENP-A nucleosome occupies a more rugged free energy landscape than the canonical H3 nucleosome. Taken together, our data suggest that CENP-A encodes enhanced distortability to the octameric nucleosome, which may allow for enhanced flexing of the histone core *in vivo*.

Histone variants are key players in the epigenetic process, encoding identity to specialized regions of the genome. CENP-A/CENH3 is a centromere specific histone H3 variant present in all eukaryotes, whose role is to dictate the single location per chromosome to which microtubules bind during every mitosis. CENP-A is thought to specify not just the location, but also structural identity to centromeres. CENP-A nucleosomes are currently shown to occupy a range of structures *in vivo*^1^,^2^,^3^,^4^,^5^,^6^,^7^,^8^,^9^,^10^,^11^,^12^,^13^,^14^,^15^,^16^,^17^,^18^. Furthermore, even *in vitro*, crystallographic and biophysical analyses conflict, with data suggesting that CENP-A octamers are more rigid than H3 octamers^10^,^19^; that CENP-A octameric nucleosomes are more compact^20^; that CENP-A nucleosomes are more unstable than H3 nucleosomes^21^; that Drosophila CENP-A can be assembled into hemisomes^15^; that yeast CENP-A nucleosomes possess a more elongated “open” conformation^11^; and finally, that human CENP-A and H3 octameric nucleosomes are essentially indistinguishable^5^,^22^,^23^. These divergent experimental observations made us curious whether subtle contributions from internal regions of CENP-A might translate to changes in the overall dynamics of the CENP-A nucleosome. To explore this possibility, we modeled CENP-A and H3 nucleosomes, as well as their respective DNA-free protein octamers, using all-atom molecular dynamics (MD) in explicit solvent.

To our surprise, despite the fact that H3 and CENP-A octameric nucleosomal crystal structures are superimposable, within 2 Å^5^, and appear almost identical by AFM^23^, computational modeling reveals distinct differences in their dynamics. CENP-A nucleosomes and octamers demonstrate greater local and global structural fluctuations than their canonical H3 counterparts. The CENP-A nucleosome reveals intrinsic local flexibility at the loop 1 region of CENP-A and increased plasticity of the CENP-A:CENP-A’ dimerization interface relative to H3:H3′. Furthermore, the CENP-A nucleosomal DNA near the pseudo-dyad is more unstable than the corresponding H3 nucleosomal DNA. Finally, our simulations demonstrate that the CENP-A nucleosome explores more conformational space than the H3 nucleosome. Taken together, our data support the possibility that pliability is an intrinsic feature of CENP-A nucleosomes, which may have implications for their structure and function *in vivo*.

## Results

**Figure 1:**
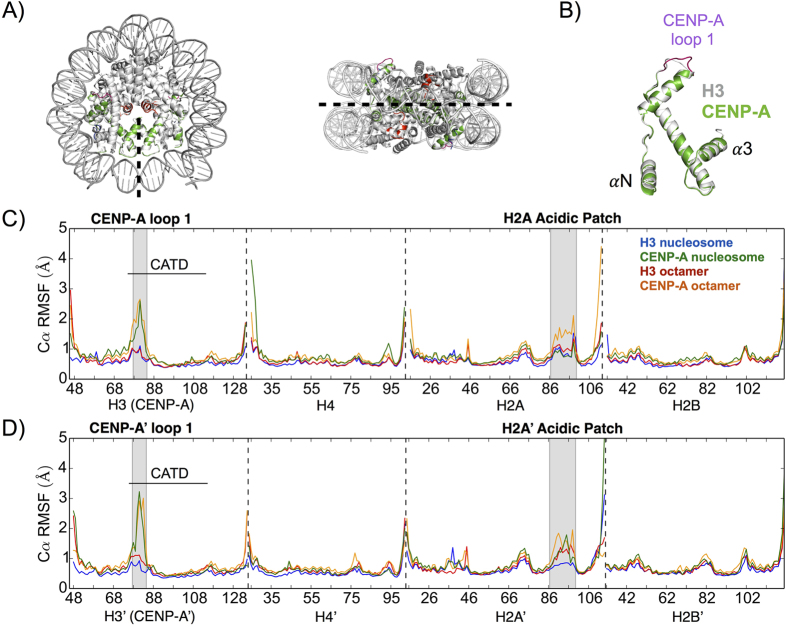
The CENP-A octamer and nucleosome structures display greater local flexibility than their canonical H3 counterparts. (**A**) The CENP-A and H3 nucleosomes are nearly identical by crystal structure alignment. Colors label CENP-A and CENP-A’ (green), CENP-A loop 1 (purple), and a region spanning the H2A acidic patch (red). The pseudo-dyad is an axis of rotational symmetry that divides the nucleosomal DNA in two and bisects the central four-helix bundle. (**B**) Structural alignment of CENP-A and H3 proteins highlights the longer CENP-A loop 1 as a major difference. (**C**) C*α* root mean square fluctuations (RMSF) of the first heterotypic half of the CENP-A and H3 structures display greater local flexibility in the CENP-A systems at several specific regions. (**D**) C*α* RMSF of the second heterotypic half demonstrates specific asymmetries in local flexibility. Dashed lines separate protein segments. Differences in C*α* RMSF greater than 0.6 Å are considered very significant. Structure figures rendered in Pymol.

Here, we explore the effects of an important histone variant, CENP-A, on the dynamics of the nucleosome. Using all-atom molecular dynamics (MD) simulations in explicit solvent on a microsecond timescale, requiring over 400,000 cpu hours in total, we investigated the structural fluctuations and dominant modes of motion of the CENP-A nucleosome in comparison to the canonical H3 nucleosome, based on the crystal structures for the octameric nucleosomes containing CENP-A (PDB ID 3AN2^5^) and H3 (PDB ID 1KX5^24^). Four systems were studied in total: (1) the canonical H3 nucleosome; (2) the octameric CENP-A nucleosome; (3) the canonical H3 octamer; and (4) the CENP-A octamer. Two copies of each histone exist within the octameric nucleosome protein cores. Following the standard nomenclature, we use a prime superscript, or absence thereof (e.g. CENP-A’ vs CENP-A), to distinguish between the two identical heterotypic halves, rotationally symmetric about the pseudo-dyad (Fig. 1A), each containing one copy of histones H3 (or CENP-A), H4, H2A and H2B.

Our analysis reveals dynamics that deviate significantly from the nearly identical crystal structures of the CENP-A and H3 nucleosomes (Fig. 1A), which are due, in part, to the significant dissimilarities in the amino acid sequences of the CENP-A and H3 proteins (Supplementary Fig. 1). Sequence and structural alignment highlights the longer loop 1 region in CENP-A compared to H3 (Supplementary Fig. 1 and Fig. 1B). We observed that it took several hundred nanoseconds for all systems to reach equilibrium. Thus, in a technical advance, we ran our simulations for a full microsecond each, in order to harvest the stable final 400 ns of each trajectory for subsequent analyses (Supplementary Fig. 2).

### The CENP-A octamer and nucleosome display greater local fluctuations than their corresponding H3 systems

We identified local structural variations within each system by computing root-mean-squared fluctuations (RMSF) of C-alphas within each system compared to their respective trajectory averages. To determine a threshold for significant differences between the local fluctuations of different systems, we calculated the RMSF of each third of the trajectories separately and obtained the standard deviation for each fluctuation. The maximum standard deviation in RMSF is ~0.3 Å, meaning any difference greater than 0.6 Å is very significant. The CENP-A loop 1 region, a region spanning the H2A’ acidic patch, and H4 *α*N helix of the CENP-A octamer and nucleosome exhibit greater structural variability than the corresponding regions for the H3 octamer and nucleosome, respectively (Fig. 1C,D).

The CENP-A loop 1 region (residues 78–84) is two residues longer than the same region of H3 and predicted to display relatively greater structural variation^5^. This region is important because of its exposure to solvent and, structurally, because it provides the connection between two major helices in H3 and CENP-A, *α*1 and *α*2. The loop 1 region is the only significance difference in local flexibility, at the individual residue level, between H3 and CENP-A proteins (Fig. 1C,D). The maximum differences in C-alpha RMSF between the loop 1 regions of CENP-A and H3 is +1.5 Å (comparing CENP-A Gly 81 and H3 Asp 81) and +2.3 Å (comparing CENP-A’ Gly 81 and H3′ Asp 81). Although they contain the same amino acid sequences, a region spanning the H2A’ acidic patch (residues 87–100) and the H4 *α*N helix (residues 26–29) display greater structural variation in the CENP-A octamer and nucleosome than in the H3 octamer and nucleosome (Fig. 1C,D). For the nucleosomal systems, we observed increased structural fluctuations only for the region spanning the H2A’ acidic patch (a maximum difference of +0.93 Å at Lys 95) and for H4 *α*N helix (a maximum difference of +2.5 Å at Ile 26), and not in their reciprocals. The observed asymmetries may occur because of the different starting points for H4 (Asn 25) and H4′ (Asp 24), and different ending points for H2A (Gln 112) and H2A’ (Val 114). The additional negatively charged residue of H4′ could discourage interactions with negatively charged DNA.

Finally, the CENP-A targeting domain (CATD, residues 75–114) has been predicted to rigidify the CENP-A/H4 interface because of experimentally measured relative increase in hydrophobicity in comparison to the H3/H4 interface^10^. However, consistent with prediction from the crystallographic evidence^5^, the only major difference in local flexibility within the CATD we observed is located at the loop 1 region. Overall, CENP-A systems appear to have greater flexibility on the local level compared to the H3 systems in three specific regions: CENP-A loop 1, a region spanning the H2A’ acidic patch, and the H4 *α*N helix.

### The CENP-A octamer and nucleosome exhibit greater global fluctuations than their H3 counterparts

**Figure 2:**
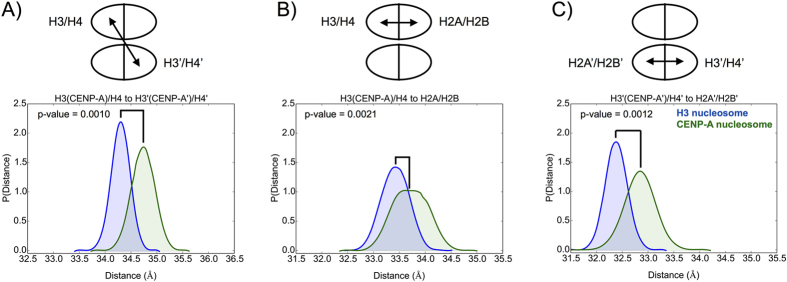
The CENP-A nucleosome exhibits greater global flexibility than the canonical H3 nucleosome. (**A**) The distance between the centers-of-mass (COMs) of the two dimers within the homotetramer is greater, on average, in the CENP-A nucleosome than in the H3 nucleosome. The distances between the COMs of the two dimers within the first (**B**) and second (**C**) heterotetramers are greater, on average, in the CENP-A nucleosome than in the H3 nucleosome. Specific average distances and standard deviations are included in Supplementary Table 1.

Beyond individual residues, structural fluctuations occur at multiple spatial scales for octamers and nucleosomes. Within CENP-A/H4 and H3/H4 dimers, our analysis demonstrates that individual CENP-A/H4 dimer pairs are on average more compact, but not more rigid, than H3/H4 in the context of either the protein octamer, or the entire nucleosome (Supplementary Fig. 3). These data are partially consistent with the prediction from experimental evidence^10^,^19^,^25^. To further investigate potential rigidity in the CENP-A nucleosome on a global scale, we determined the distances between the centers-of-mass (COM) of dimers in the homotetramer (Fig. 2A), and between dimers in the two heterotetramers (Fig. 2B,C, Supplementary Table 1, and Supplementary Fig. 4). We find that the distance between CENP-A/H4 dimers is greater than between H3/H4 dimers in both the octamer structures (34.5 ± 0.29 Å in the CENP-A octamer; and 33.9 ± 0.21 Å in the H3 octamer) and nucleosome structures (34.7 ± 0.22 Å in the CENP-A nucleosome; and 34.3 ± 0.18 Å in the H3 nucleosome). This difference is more evident in the absence of DNA. The average distance between H3 (CENP-A)/H4 and H2A/H2B dimers is virtually identical, with a greater standard deviation, for the CENP-A octamer (33.6 ± 0.39 Å) than for the H3 octamer (33.7 ± 0.25 Å). The same average distance is greater for the CENP-A nucleosome (33.7 ± 0.34 Å) than for the H3 nucleosome (33.4 ± 0.27 Å). The distance between dimers for the second heterotetramer, between H3′ (CENP-A’)/H4′ and H2A’/H2B’, is slightly greater for the CENP-A octamer (33.0 ± 0.35 Å in the CENP-A octamer; and 32.8 ± 0.27 Å in the H3 octamer) and significantly greater for the CENP-A nucleosome (32.8 ± 0.30 Å in the CENP-A nucleosome; and 32.4 ± 0.21 Å in the H3 nucleosome) compared to their canonical counterparts. In total, these data indicate that the CENP-A octamer and nucleosome display greater fluctuations on a global level than the H3 octamer and nucleosome. The greater distances observed between histone dimers within the CENP-A nucleosome, compared to the canonical H3 nucleosome, are consistent with recently published work^26^, which, using fluorescent probes to measure the distance between specifically defined regions of H2B histones, reports that histones in the centromeric nucleosomes are more loosely packed than in the canonical H3 nucleosome (between H2B and H2B’).

### Greater global fluctuations are underpinned by weaker contacts at the CENP-A:CENP-A’ dimerization interface

**Figure 3:**
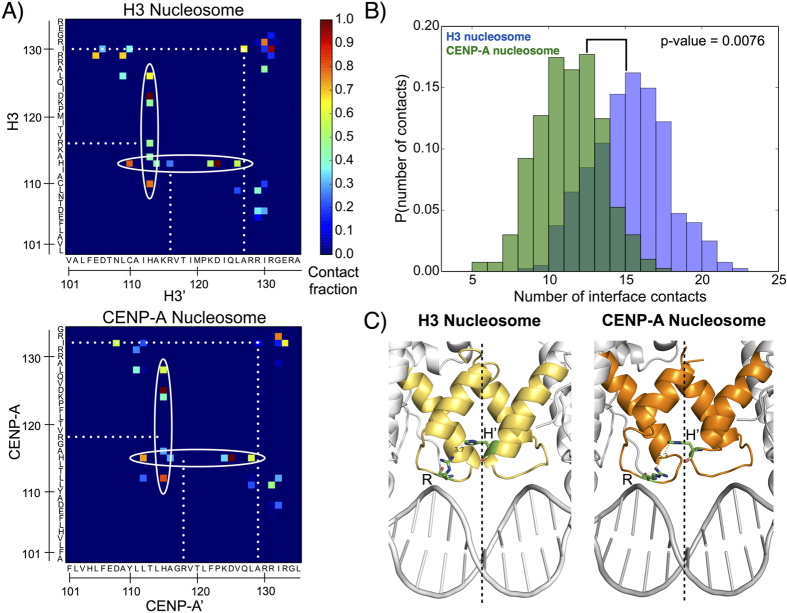
The CENP-A dimerization interface forms fewer and weaker contacts than the corresponding H3 interface. (**A**) Contact map analysis illustrates the promiscuous interactions of His113/115 (circled) and contacts present in the dimerization interface of the H3 nucleosome but not in the corresponding interface of the CENP-A nucleosome (dotted lines). (**B**) Histograms demonstrate that the number of contacts formed at the CENP-A dimerization interface is fewer, on average, than at the corresponding H3 interface. (**C**) Crystal structure dimerization interfaces for the CENP-A (dark orange) and H3 (light orange) nucleosomes highlight that the four helices composing the CENP-A:CENP-A’ interface are less well-defined than the four helices composing H3:H3′. Residues Arg 116 and His’ 113 of H3:H3′, as well as Arg 118 and His’ 115 of CENP-A:CENP-A’, are shown as sticks. The dashed lines represent the pseudo-dyads. Structure figures rendered in Pymol.

One plausible reason for the global fluctuations seen above might be increased flexibility in the CENP-A dimerization interface relative to the corresponding interface of H3. To test this hypothesis, we performed contact map analysis to compare the specific contacts that form the H3:H3′ and CENP-A:CENP-A’ interfaces (Fig. 3A,B). Quantitatively, 4 contacts out of the 15 formed by H3:H3′ are lost in the CENP-A:CENP-A’ interface (Fig. 3B), and 6 are weakened. For instance, His113/115 is a key residue in the crystal structures of both octameric nucleosomes, binding to Asp123/125^27^. In our simulations, this contact is still prominent, however, His113/115 is promiscuous, making multiple contacts at the dimerization interface. These contacts in the CENP-A:CENP-A’ interface are, on average, weaker than the corresponding set in H3:H3′ (Fig. 3A), and several contacts are lost altogether. Another clear difference is the contacts formed by H3 His 113 and H3′ Arg 116, and by the reciprocal set of interactions. The corresponding contacts are not present between CENP-A and CENP-A’. This difference is due, in part, to shorter, less well defined, helices composing the four helix bundle of the CENP-A:CENP-A’ interface compared to H3:H3′ (Fig. 3C) and a longer minimum distance between CENP-A Arg 118 and CENP-A’ His 115 (5.3 Å) than between H3 Arg 116 and H3′ His 113 (3.7 Å). Furthermore, two hydrophobic interactions present between H3 and H3′ (Ile 130 to Ala’ 137, and its reciprocal) are lost between CENP-A and CENP-A’. We observed a similar contrast between the H3:H3′ and CENP-A:CENP-A’ dimerization interfaces in the octameric structures (Supplementary Fig. 5). Generally, our analysis demonstrates that the CENP-A:CENP-A’ dimerization interface is weaker than that of H3:H3′, suggesting that the CENP-A:CENP-A’ interface could exhibit greater plasticity.

### Greater plasticity at the CENP-A:CENP-A’ dimerization interface is a dominant mode of motion

**Figure 4:**
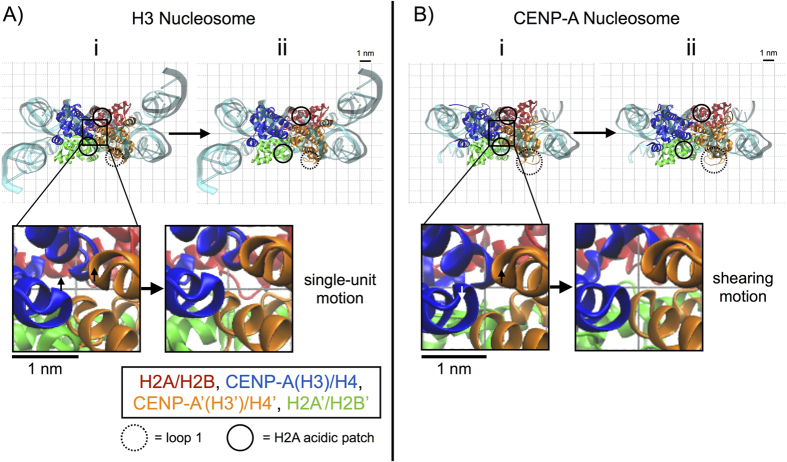
Increased plasticity of the CENP-A dimerization interface is a dominant mode of motion. (**A**) 2D projection of the top principal component of the H3 nucleosome onto the representative structure, viewed from the side of the DNA pseudo-dyad, highlights mobility in the H2A’ acidic patch restrained by H3′ and a lack of movement in H3′ loop 1. The inset displays the single-unit motion of the H3 dimerization interface. (**B**) 2D projection of the top principal component onto the representative structure for the CENP-A nucleosome highlights mobility in the H2A’ acidic patch unrestrained by CENP-A’ and enhanced fluctuations in CENP-A’ loop 1. The inset displays the shearing and pinching motion of the CENP-A dimerization interface. Structure figures rendered in VMD.

We tested the possibility of increased plasticity in the CENP-A dimerization interface by performing principal component analysis (PCA), which determines the dominant modes of motion for each system. Projections of the top principal components from C*α* PCA onto representative structures in 2D plots (Fig. 4 and Supplementary Fig. 6) and 3D movies (Supplementary Movies 1 and 2) illustrate the magnitude and direction of dominant motions for the H3 and CENP-A structures. These principal components clearly contrast the natures of the motion associated with the H3:H3′ and the CENP-A:CENP-A’ dimerization interfaces. Interestingly, in the top two principal components for the H3 nucleosome, H3 and H3′ move together as a single unit in a concerted manner, maintaining the integrity of their interface (Fig. 4A and Supplementary Movie 1). In contrast, in the first principal component of the CENP-A nucleosome, CENP-A and CENP-A’ visibly move separately from each other in a shearing motion (Fig. 4B and Supplementary Movie 2). Furthermore, in their respective top principal components, the CENP-A nucleosome exhibits greater breathing motion (a global opening and closing of the central void) than the H3 nucleosome (Supplementary Movies 1 and 2), and the four helices defining the CENP-A:CENP-A’ interface display anti-correlated pinching motions (Supplementary Movie 2). We observed a similar contrast between the dimerization interfaces of the H3 and CENP-A octamers. In their respective first two principal components in the octamer structures, H3 and H3′ move together at their interface (Supplementary Fig. 6A) and CENP-A and CENP-A’ move away and towards each other at their interface (Supplementary Fig. 6B). Overall, the PCA analysis indicates that a weakened interface drives greater plasticity at the CENP-A:CENP-A’ dimerization interface within the CENP-A nucleosome.

### H2A patch mobility is sensitive to surrounding local interactions

The acidic patch is a unique structural motif of the nucleosome surface, carrying the greatest net charge of the solvent-exposed region of the histone octamer surface^27^,^28^. Formally defined by eight residues (H2A E56, E61, E64, D90, E91, E92 and H2B E102, E110), the acidic patch forms a complex interface, with a high negative charge density and distinct groove shape. Furthermore, the acidic patch is topologically poised to interact with multiple types of chromatin factors, including the H4 N-terminal tail of an adjacent nucleosome^27^, Sir3^29^, LANA^30^, and the CENP-A specific CENP-C^31^, essential in forming the inner kinetochore. From our investigation of local flexibility, we identified an important region of H2A (residues 87–100) spanning the H2A C-terminal extension part of the acidic patch (H2A D90, E91, and E92). This region contains a short *α*-helix bracketed between H2A *α*3 and the long H2A C-terminal tail. Importantly, the region spanning the H2A acidic patch undergoes similar structural fluctuations in the first heterotypic half of the CENP-A and H3 nucleosomes (Fig. 1C) and greater structural variation in the CENP-A nucleosome than in the H3 nucleosome in the second heterotypic half (Fig. 1D). We first examined the results from principal component analysis to investigate this asymmetric behavior.

**Figure 5:**
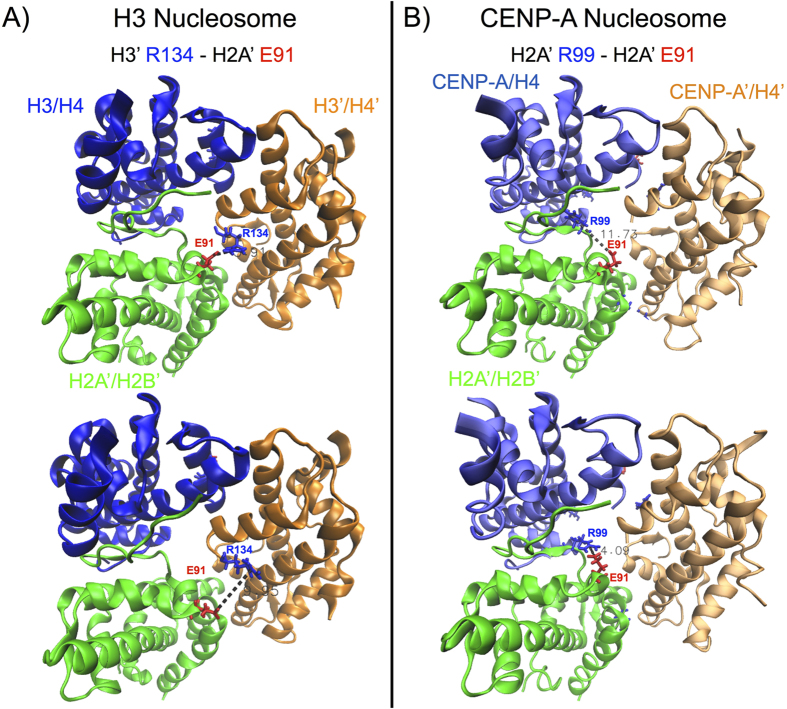
H2A’ acidic patch mobility depends on local, electrostatic interactions. Representative simulation snapshots highlight electrostatic interactions contributing to the mobility of a region spanning the H2A’ acidic patch in (**A**) the H3 nucleosome, between H3′ Arg134 and H2A’ Glu91, and in (**B**) the CENP-A nucleosome, between H2A’ Arg99 and Glu91. Colored sticks represent positive residues (in blue) and negative residues (in red). Dashed lines measure the distances between the center of positive charge on the arginines, CZ, and the center of negative charge on the glutamic acid, CD. The surrounding superhelical DNA and the H2A/H2B dimers are removed to facilitate observation. Structure figures rendered in VMD.

The PCA analysis revealed differences in the modes of motion of the CENP-A and H3 systems in addition to those found at the dimerization interfaces. We observed structural fluctuation in the H2A’ acidic patch of the H3 and CENP-A nucleosomes in the top principal components, but the natures of these fluctuations are very different (Fig. 4). For the H3 nucleosome, the H2A’ acidic patch moves together with the H3′ C-terminus, a salt bridge between H3′ Arg 134 and H2A’ Glu 91 playing a contributing factor (Fig. 4A and Supplementary Movie 1). In contrast, in the first principal component of the CENP-A nucleosome, the H2A’ acidic patch moves up and down the DNA supercoil axis, and into and out of the central void, independently of the CENP-A’ C-termini (Fig. 4B and Supplementary Movie 2), which lacks the positively charged Arginine seen in H3 (i.e. Arg 134). Therefore, PCA analysis provides insight into the mobility of the region spanning the H2A’ acidic patch in the CENP-A nucleosome. The top principal components of octameric structures indicate a similar increase in the overall mobility of the region spanning the H2A’ acidic patch for the CENP-A system compared to H3 in the absence of DNA (Supplementary Fig. 6). Because of the asymmetry between structural fluctuations of the regions spanning the H2A and H2A’ acidic patches (Fig. 1C vs 1D), we decided to investigate further by extending our MD simulation by 400 ns with reduced position restraints (reduced from K = 2.5 × 10^−1^ kJ/(mol nm^2^) to K = 5.0 × 10^−5^ kJ/(mol nm^2^)). We concluded that the H2A acidic patch occupies a rugged conformational landscape, and the overall mobility depends on transient, local interactions. We focused on the H2A’ acidic patch, where a significant and variable difference exists between the CENP-A and H3 nucleosomes (Supplementary Fig. 7). We identified specific, local electrostatic interactions that contribute to H2A’ acidic patch mobility in the H3 nucleosome (between H3′ R134 and H2A’ E91, Fig. 5A) and in the CENP-A nucleosome (between H2A’ R99 and E91, Fig. 5B). The stochastic formation and disruption of these interactions, among others, can alternatively restrain and relax the H2A’ acidic patch, demonstrating that its mobility is highly sensitive to the dynamic rearrangement of local interactions.

### CENP-A nucleosomal DNA near the pseudo-dyad is relatively unstable compared to the corresponding H3 nucleosomal DNA

We were curious whether the local and global fluctuations in the CENP-A nucleosome noted above (Figs 1, 2, 3, 4) had an impact on the DNA wound about the CENP-A nucleosome. Past experiments have shown that CENP-A protects ~120 bp of DNA relative to H3, which almost exclusively protects 147 bp of DNA. The difference in binding has been attributed to missing Arginines in the *α*N helices of CENP-A^21^. Recent computational short-timescale simulations have suggested the exit/entry helices of DNA have weaker interactions with the CENP-A protein core^32^. Exit and entry DNA “site exposure” is an important biological feature^33^, and in the context of the CENP-A nucleosomes, could potentially destabilize H2A/H2B binding, resulting in losing this dimer pair from the octamer, thus destabilizing the nucleosome.

In our long-timescale simulations, we noted that the entry/exit fluctuations of DNA are similar around the H3 and CENP-A nucleosome, with roughly equal propensity for local and asymmetric unwinding. The H3 nucleosome displays asymmetric site exposure, freeing up ~15 bp of the exit nucleosomal DNA consistent with recent biochemical experiments suggesting asymmetric behavior in the unwinding of H3 nucleosomal DNA *in vivo*^34^ and *in vitro*^35^. ~15 bp of the entrance nucleosomal DNA becomes completely exposed in the CENP-A nucleosome, the detachment occurring at the opposite side to the H3 nucleosome. It should be noted that this type of entry/exit detachment event could be stochastic in nature, because *in vivo*, DNA traces a continuous path via the linker DNA to adjacent nucleosomes in the array, and the presence or absence of proteins such as H1 (for the H3 nucleosome), CENP-C and CENP-B (for the CENP-A nucleosome), will likely alter the stability and crossing-over of exit and entry DNA. Thus, asymmetric unpeeling of the palindromic (i.e. symmetric) *α*-satellite based DNA sequences resolved in the crystal structures could be a probabilistic event, in part, governed by structural motifs that may exist within the centomeric DNA sequences *in vivo*^34^,^35^. Furthermore, the asymmetric unpeeling observed in our simulations could be an example of spontaneous symmetry breaking that is stochastically induced by structural fluctuations, either locally or at a large scale.

**Figure 6:**
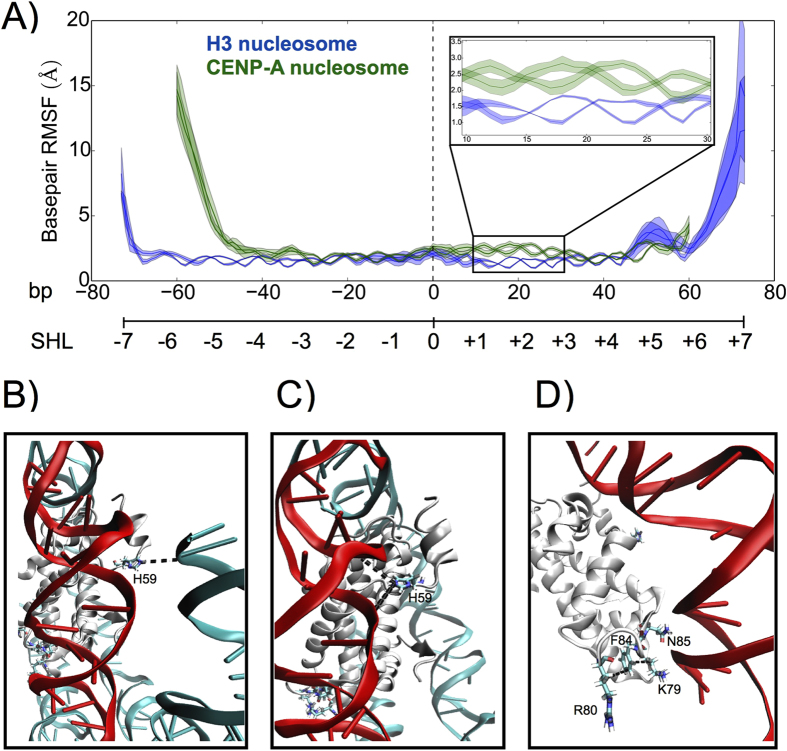
CENP-A nucleosomal DNA is relatively unstable near the pseudo-dyad compared to the corresponding H3 nucleosomal DNA. (**A**) RMSF per basepair for the CENP-A and H3 nucleosomes demonstrates asymmetric entry/exit unwinding for both systems and further highlights significantly increased fluctuations for CENP-A basepairs +10 to +30 (inset). Two lines are shown for each system since the DNA is double-stranded, and the shaded areas represent the RMSF ± one standard deviation. (**B**) His 59, unique to CENP-A, has electrostatic interactions with the entry terminal of DNA before it detaches. (**C**) Coinciding with the detachment of entry DNA, His59 turns towards DNA SHL +1 or +2, disrupting the local stability of DNA. (**D**) N85 in CENP-A, substituted for R83 in H3, has a weaker binding affinity to DNA, and two other substitutions, F84 and R80, develop a hydrophobic hotspot with K79 of H4, further contributing to DNA instability near the pseudo-dyad. Basepairs 10–30 are highlighted in red, key residues are shown as sticks, and CENP-A interactions not found in the H3 nucleosome are represented by dashed lines. Structure figures rendered in VMD.

Significantly, in our long timescale simulations above (Fig. 6), we observed that CENP-A nucleosomal DNA exhibits greater structural fluctuations near the pseudo-dyad, from SHL +1 to +3, which extrude away from the octameric core surface. Throughout these 20 bp of DNA, the CENP-A DNA basepair fluctuations were on average ~1.0 Å greater than the corresponding base pairs in the H3 nucleosome (Fig. 6A inset). We performed event coincidence analysis (Fig. 6B–D and Supplementary Fig. 8) to unveil factors contributing to the release of ~20 bp pseudo-dyad proximal DNA. We tracked several key residues in CENP-A and H4, finding an increase in the distance between CENP-A H59 (a substitution for E59 in H3) and the entry helix of nucleosomal DNA at bp −60 (Supplementary Fig. 8.A) upon detachment. This stochastic event coincides with CENP-A’ H59 swinging inwards to DNA SHL +1 or +2. This swing disrupts the local interactions between CENP-A’ and DNA, including CENP-A’ K64 and bp +19, causing the ripple of DNA moving away from the protein core. H59 (a positive residue) in CENP-A replaces E59 (a negative residue) of H3. H59 is located near the N-terminus of CENP-A, and is important for the stability of CENP-A nucleosomal entry DNA, forming electrostatic interactions with the negatively charged DNA (Fig. 6B). Once this section of DNA detaches from the CENP-A protein octamer, CENP-A H59 is available to form alternating interactions between two pseudo-dyad proximal turns of DNA (Fig. 6C and Supplementary Fig. 9). These interactions alone can increase the base pair structural fluctuations because they are not found in the native CENP-A crystal structure. H59 and K64 compete for the same interaction with DNA, both positive residues naturally repel each other, and both favor similar electrostatic interactions with the highly negatively charged DNA. The replacement of H3 E59 with CENP-A H59 is definitely an important factor in CENP-A nucleosomal DNA flexibility near the pseudo-dyad, however, it is not the only factor. This ripple is amplified by an intrinsically weak interaction of this section of DNA because of a missing R83 present in H3, but not in CENP-A. Finally, a substitution of L82 (present in H3) by a F84 in CENP-A, creates a hydrophobic hotspot with CENP-A R80 and H4 K79 (Supplementary Fig. 8B,C), weakening K79′s affinity to DNA. Common contacts between two arginine residues, counterintuitive from an electrostatic perspective, have been investigated both from the vast number of experimentally determined crystal structures within the Protein Data Bank^36^, and by molecular dynamics simulation^37^,^38^. Arginine and lysine residues both have long side chains, which feature a positively charged head group and a hydrophobic tail. Examining Fig. 6D in close detail, we observe that F84 (a hydrophobic residue) interacts with the long tail regions of R80 and K79, both of which are hydrophobic. The accumulation of CENP-A DNA endpoint detachment, CENP-A H59 replacing H3 E59, and the formation of a hydrophobic hotspot within the CENP-A nucleosome conspire to release ~20 bp of DNA from the pseudo-dyad proximal region of CENP-A nucleosome.

### The CENP-A nucleosome features a more rugged free energy landscape than the canonical H3 nucleosome

**Figure 7:**
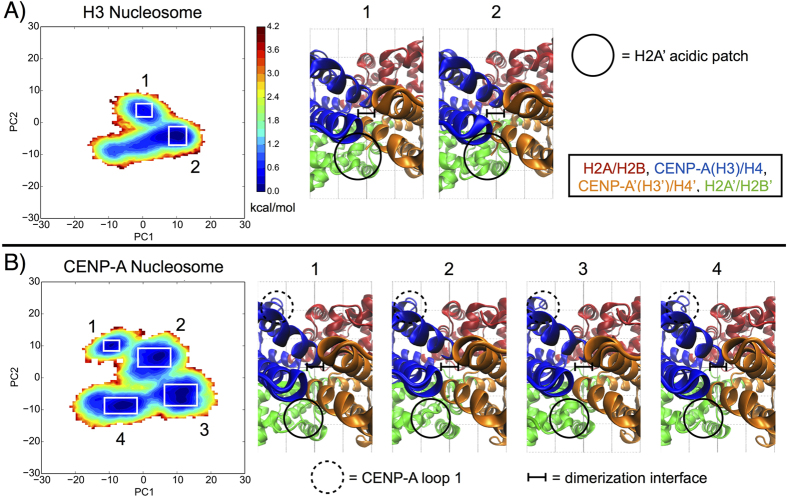
The characteristic free energy landscape is more rugged for the CENP-A nucleosome than for the H3 nucleosome. (**A**) Free energy projection of the H3 nucleosome onto its first two principal components reveals two distinct conformational basins. (**B**) Free energy projection of the CENP-A nucleosome reveals four distinct conformational basins. White boxes highlight distinct free energy basins. Insets highlight key differences between representative structures for the numbered basins, consistent with the rest of our analysis. Structure figures rendered in VMD.

We compared the conformational space explored by the nucleosome systems by projecting the trajectories onto their corresponding top two principal components, thereby mapping two-dimensional free energy landscapes (Fig. 7)^39^,^40^. The landscape topography of the canonical H3 nucleosome contains broad and well-connected basins, whereas the number of basins, and the barriers between basins, is greater for the CENP-A nucleosome. When examining representative structures of the free energy basins for the H3 nucleosome, we observe different conformations for the H2A’ acidic patch, concordant with differences in the H3′ C-terminus (Fig. 7A). In contrast, there are multiple conformations for the CENP-A:CENP-A’ dimerization interface in the distinct basins for the CENP-A nucleosome, featuring different arrangements of the four helices defining this interface (Fig. 7B). Representative structures for the CENP-A nucleosome also display different conformations for CENP-A loop 1 and the H2A’ acidic patch. Specifically, the H2A’ acidic patch is more disordered in CENP-A representatives 1 and 2, and more ordered in representatives 3 and 4 (Fig. 7B). Globally, the CENP-A nucleosome free energy landscape covers more conformational space (i.e. greater overall area) than the H3 nucleosome free energy landscape. From our two-dimensional free energy landscapes of the CENP-A and H3 nucleosomes, we calculated the average free energy barriers between distinct conformational basins. For the CENP-A nucleosome, we determined the free energy barriers between adjacent, and accessible, conformational basins to be ~2 to 4 *k*_*B*_*T*, and for the single free energy barrier for the H3 nucleosome to be ~2 *k*_*B*_*T*. This calculation illustrates that the CENP-A nucleosome occupies a more rugged free energy landscape, and that both CENP-A and H3 nucleosomes can overcome free energy barriers to convert between different conformational basins. Overall, the CENP-A nucleosome has more distinct conformational basins than the H3 nucleosome, and the structural differences between free energy basins correspond to the contrasts in local and global fluctuations we observed through other modes of analysis.

## Discussion

Here, we present the first microsecond timescale all-atom computational investigation of the conformational dynamics of canonical H3 and CENP-A variant octamers and nucleosomes in explicit solvent. This significantly extended timescale relative to previous explicit solvent all-atom studies of nucleosomes containing H3^41^,^42^,^43^ and CENP-A^32^ allowed our analyses to reveal the internal protein dynamics of the H3 and CENP-A nucleosomes at stable equilibrium (Supplementary Fig. 2). For H3, we observe consistency with previously published experimental results on the stability of the DNA near the pseudo-dyad (Fig. 6)^24^,^27^,^44^,^45^, and asymmetric unwinding of the entry and exit DNA (Fig. 6)^35^, and with previous computational studies identifying structural mobility in H2A (Fig. 4)^43^ and flexibility at the entrance and exit DNA (Fig. 6)^41^. For CENPA, we also observe consistency with previously published results and predictions of flexible DNA edges (Fig. 6)^5^,^6^,^11^,^21^,^23^,^32^,^46^,^47^,^48^,^49^,^50^,^51^, greater structural variation in CENP-A loop 1 relative to the same region of H3 (Fig. 1), predicted experimentally^5^ and observed computationally^32^, as well as increased CENP-A/H4 dimer compactness relative to H3/H4 (Supplementary Fig. 3)^25^. However, using long timescale simulations coupled to principal component analyses and free energy landscape theory, our data suggest that the CENP-A nucleosome is structurally flexible on local and global scales.

Most importantly, the overall increase of global flexibility in the CENP-A nucleosome (Fig. 2) is underpinned by a reduced number of contacts in the CENP-A:CENP-A’ dimerization interface (Fig. 3). This results in the CENP-A:CENP-A’ four helix bundle undergoing a distinctive and unique shearing motion coupled to a pinching motion (Fig. 4 and Supplementary Movie 2). This motion translates to a flexing of the entire CENP-A nucleosomal and octameric particles, such that CENP-A containing complexes explore significantly more rugged energy landscapes (Fig. 7 and Supplementary Fig. 10). A major consequence of this rugged landscape is additional free energy minima are available to the CENP-A nucleosome, which can spontaneously visit these basins with near equal probability. Plasticity in the CENP-A dimerization interface is driven by the dynamic rearrangement of mostly weak contacts between the *α*2 and *α*3 helices of the CENP-A proteins (Fig. 3). In addition to the CENP-A dimerization interface, several other specific regions contribute to the greater overall flexibility of the CENP-A nucleosome compared to the H3 nucleosome.

First, we note that the mobility of the region spanning the acidic patch of the H2A depends on the surrounding local interactions in the CENP-A and H3 nucleosomes (Figs 1 and 5). The stochastic formation and disruption of electrostatic interactions at the interface of the H2A acidic patch and other histones, and within the H2A acidic patch, can alternatively restrain and relax this region (Fig. 5). Variable mobility of H2A in the CENP-A could effect its ability to interact freely with non-histone proteins, such as the kinetochore protein CENP-C, which has experimentally been shown to bind to the acidic patch of H2A in the context of a hybrid CENP-A fusion nucleosome^52^. In addition, the highly flexible CENP-A loop 1 (Figs 1 and 4B) could play an important regulatory role in the formation of higher order CENP-A chromatin^53^. Furthermore, CENP-A nucleosomal DNA displays asymmetric instability near the pseudo-dyad, relative to H3 nucleosomal DNA (Fig. 6 and Supplementary Fig. 9). One predicted outcome of such instability near the pseudo-dyad is increased access to the protein octameric core. These data could provide a potential explanation for experimentally observed acetylation seen within the CENP-A nucleosomal core at H4 K79 *in vivo*^14^.

Lastly, these *in silico* findings have important implications for the behavior of CENP-A nucleosomes *in vitro* and *in vivo*^6^,^7^,^9^,^11^,^19^. Specifically, our data shows the loss of DNA contacts near the pseudo-dyad (Fig. 6D), a weaker CENP-A dimer interface (Fig. 3), coupled to shearing of the four-helix bundle (Fig. 4). Even in the DNA-free CENP-A octameric core, we observe global shearing motions between the two CENP-A/H4/H2A/H2B heterotetramers (Supplementary Fig. 4A).

So far, there is no direct experimental evidence testing whether or how octameric nucleosomes can unpeel at the pseudo-dyad to generate two hemisomes^9^,^54^. However, based on these results, it is plausible that CENP-A hemisomal intermediates might reflect increased distance and weaker contacts between two heterotypic halves of the octameric CENP-A nucleosome, coupled to looser DNA contacts at pseudo dyad proximal region, both of which could be exaggerated by specific biological conditions *in vivo*. Our simulations of DNA-free octameric CENP-A particles do not appear to show striking disruptions of H2A/H2B in the octameric core, as would be predicted from a hexameric structure^7^. However, we note that H2A/H2B have slightly increased distance from CENP-A/H4 in the CENP-A nucleosome (Fig. 2). Thus, as has recently been noted for H3 nucleosomes^34^,^55^, it is possible that stochastic asymmetric entry/exit DNA site exposure, coupled to chromatin remodelers such as RSF^56^, could effect the eviction of one or both H2A/H2Bs in the CENP-A nucleosome.

It has already been demonstrated that *in vitro*, and *in vivo*, internal covalent modifications of H3 can alter canonical nucleosomal conformation and stability^57^,^58^,^59^,^60^, which may utilize alternative modes of internal motion relative to the motions described for CENP-A in this study. The data presented above support the possibility that *in vivo* CENP-A nucleosomes subjected to pulling, pushing or twisting mechanical forces may geometrically adapt to extrinsically imposed deformations by exploiting internal pliability. Such conformational changes in the CENP-A nucleosome are likely to be promoted or prohibited by specific inner kinetochore proteins like CENP-C, or specific modifications, which may predominantly favor one conformational basin of CENP-A over another at specific points of the cell cycle, or in response to one or more binding partners^31^. Consequently, interrogating the effects of biological forces, covalent modifications, point and domain mutations, and the binding of kinetochore and non-kinetochore proteins on the stability of CENP-A nucleosomes are critical and exciting future avenues of research awaiting investigation. It is feasible that internal flexibility within the CENP-A octameric nucleosome may permit exploration of multiple conformations, contributing to CENP-A’s structural and epigenetic signature *in vivo*.

## Methods

### Simulation protocol

We performed all-atom molecular dynamics (MD) using the gromacs 4.5.7 MD software^61^, the amber99SB^*^-ILDN^62^,^63^ force field for proteins, the parmbsc0^64^ force field for DNA, the ions94^65^ force field for ions, and the TIP3P water model. Starting from crystal structures for the canonical H3 nucleosome (PDB ID: 1KX5)^24^ and the CENP-A nucleosome (PDB ID: 3AN2)^5^, we built models for four systems: (1) the canonical H3 nucleosome; (2) the CENP-A nucleosome; (3) the canonical H3 histone protein octamer; and (4) the CENP-A histone protein octamer.

The following modifications were made to the experimentally determined crystal structures for a fair comparison. Canonical histone lengths were redefined to match the tailless histones found in the CENP-A crystal structure (3AN2). The missing section for CENP-A’ chain E, Thr 79 to Asp 83, was generated with MODELLER, using the corresponding region in CENP-A chain A as a homologous structure. Lastly, non-standard Mse residues found in the crystal structures were replaced with Met (a single atom substitution, Se to S).

We used the *pdb2gmx* tool in gromacs to set the Lys and Arg residues to +1e, the Asp and Glu residues to −1e, the Gln residues to neutral, and to protonate the His residues solely at NE2. Each system was solvated in a rectangular water box, ensuring a minimum buffer length of 15 Å between the system and the edges of the box. We introduced Na^+^ and Cl^−^ ions to neutralize the charge and represent the physiological 0.150 M NaCl environment. The systems were minimized using steepest descent, until reaching a maximum force less than 100 kJ/(mol nm). Periodic boundary conditions were employed throughout all the simulations, and long-range electrostatics were treated with the Particle Mesh Ewald method^66^. Non-bonded Coulomb and Lennard-Jones interactions were truncated at 10 Å, and all bonds involving hydrogen were constrained using the LINCS^67^ algorithm. After minimization, the systems were heated to 300 K by 500 ps of protein and DNA restrained NVT MD simulation followed by 500 ps of NVT MD simulation with weak harmonic restraints on protein and DNA atoms (K = 2.5 × 10^−1^ kJ/(mol nm^2^)). Weak restraints were used throughout the simulations after the initial protein and DNA restrained NVT simulation in order to prevent large-scale translation and rotation because of the non-cubic water boxes. After reaching thermal equilibrium, the systems were equilibrated at 300 K and 1.0 bar for 1.5 ns in the NPT ensemble.

To characterize the structure and dynamics of the canonical and CENP-A nucleosomes and octamer cores, we performed production all-atom MD simulations in the NPT ensemble at 1.0 bar and 300 K with a 2 fs time-step, saving coordinates, velocities, and energies every 2 ps for further analysis. We updated the list of non-bonded neighbors every 10 steps. Using the V-rescaled, modified Berendsen thermostat^68^ with a 1.0 ps time-constant and the Parrinello-Rahman barostat^69^ with a relaxation time of 2.0 ps, we performed one microsecond of MD simulations, only considering the final 400 ns for analysis, with weak position restraints on heavy atoms (K = 2.5 × 10^−1^ kJ/(mol nm^2^)) for each system. These restraints do not interfere with common internal motions, both local and collective, but could repress unusually large-scale displacements or major structural disruptions. Hence, we ran an additional 400 ns of MD simulations with significantly reduced position restraints (K = 5.0 × 10^−5^ kJ/(mol nm^2^)), which do not interfere with internal collective dynamics at any length scale. Performing the same analysis for the two above-mentioned restraint values, CENP-A and canonical H3 structures were found to undergo the same characteristic local and global dynamics except for the H2A’ acidic patch. Therefore, we separately addressed the sensitivity of H2A acidic patch mobility to the surrounding local interactions.

It is important to consider the choices made in designing our models, and future directions moving forward with all-atom MD simulations. Here, we do not include the tail domains in our models because they are not resolved in the octameric CENP-A nucleosome (PDB ID: 3AN2^5^), and we want to make the comparison between the CENP-A and canonical H3 structures as fair as possible. The intrinsically disordered tail regions will have important effects, however, these effects will depend heavily on the initially determined conformations. From a simulation standpoint, each tail adopts an ensemble of conformations, and 1 *μ*s of constant *T* all-atom MD simulations in explicit solvent would not provide sufficient sampling to reach equilibrium. The most recent canonical H3 nucleosome crystal structure (PDB ID: 1KX5^24^) used special chemical additives to stabilize the tail regions enough for x-ray diffraction. Different computational techniques, such as replica exchange molecular dynamics, are often used to investigate histone tail regions in order to enhance sampling, overcoming the many barriers in the rugged free energy landscapes such tails occupy^70^,^71^,^72^,^73^. Examining the specific role of histone tails within an individual nucleosome is an important future direction, upon the publication of an experimentally resolved octameric CENP-A nucleosome crystal structure that includes histone tail domains.

The possible effects of DNA length and sequence on nucleosome dynamics are important to consider as well. For the results shown in the main text, we only consider the central 121 base pairs of DNA for the CENP-A nucleosome, since only these base pairs, from a 147 base-pair long sequence, were visible in the CENP-A nucleosome crystal structure (PDB ID: 3AN2^5^). The arrangement of these additional base pairs of CENP-A nucleosomal DNA is not known for sure, i.e. it is not clear that they follow the same superhelical path observed experimentally for the canonical H3 nucleosome. Indeed, Tachiwana *et al.*^5^ hypothesize that the terminal regions of DNA could be more flexible than the corresponding regions in the canonical H3 nucleosome due to structural differences between CENP-A and H3.

We performed another simulation transplanting the experimentally resolved 147 base pairs of canonical H3 nucleosomal DNA (from PDB ID: 1KX5^24^) onto the CENP-A nucleosome. In the Supplementary Information, we compare CENP-A nucleosome dynamics with both the 121 bp from 3AN2^5^ (“CENP-A^121^”) and with the 147 bp from 1KX5^24^ (“CENP-A^147^”) to the canonical H3 nucleosome^24^ (“H3^147^”), considering the CENP-A dimerization interface (Supplementary Fig. 11A), the distances between histone dimers (Supplementary Fig. 11B), and nucleosomal DNA flexibility (Supplementary Fig. 11C). These data show a potential increase in the stability of interactions between CENP-A/H4 and H2A/H2B in the first heterotypic half and in the stability of CENP-A nucleosomal DNA near the pseudo-dyad when 13 bp are added to entry and exit regions of DNA (Supplementary Fig. 11B,C). However, the interactions of the internal CENP-A/H4 homotypic core remained similar (Supplementary Fig. 11A,B).

### Analysis of the trajectories

We first determined the root-mean-square fluctuations (RMSF) of every C*α* atom for all of the studied systems. C*α* RMSF serves as a measure of the local structural fluctuations, as compared to the geometric average structure, identifying specific residues, and protein regions, that exhibit greater flexibility. We compared the distances between the centers-of-mass (COM) of the H3 (and CENP-A) *α*1 helix and H4 *α*2 helix to provide insight into the structural fluctuations within one protein dimer. Furthermore, we examined the global structure and dynamics of the canonical and CENP-A systems by comparing the distances between dimers within histone tetramers. Four dimers provided coarse-grained definitions for the protein component of the canonical, H3 systems (H3/H4, H2A/H2B, H3′/H4′, H2A’/H2B’) and of the CENP-A systems (CENP-A/H4, H2A/H2B, CENP-A’/H4′, H2A’/H2B’). We analyzed inter-residue contact preferences at the interfaces of H3:H3′ and CENP-A:CENP-A’. A contact was determined to exist when the distance between two non-hydrogen atoms from different residues was less than 3.6 Å. Contacts were calculated as fractions of time of their respective entire trajectories. This contact definition will include hydrogen bonds, electrostatic interactions, and hydrophobic interactions between residues, without distinguishing the specific type of contact. In general, the length scales defining electrostatic and hydrophobic interactions are longer than those defining hydrogen bonds. For example, distances between oppositely charged heavy-atoms from different residues under 4.0 Å is a common salt-bridge definition^74^. We investigated DNA structural fluctuations by calculating basepair RMSF for the CENP-A and H3 nucleosomal DNA, and the associated standard deviations were determined by the contributions of each third of the trajectory to the structural variation in each basepair.

Principal component analysis (PCA) was performed to extract the dominant modes of motion of the nucleosomal and octameric structures from the MD simulation trajectories^75^. Overall translational and rotational motion in the trajectories were eliminated by a translation to the average geometric center and by alignment to the energy-minimized structure. Using the Cartesian coordinates of all the C*α* atoms (*N* = the number of C*α* atoms), we generate a 3*N* × 3*N* covariance matrix. The diagonalization of this matrix provides a set of eigenvectors that give a vectorial description of each component of motion. Every eigenvector has a corresponding eigenvalue that represents the contribution of that component of motion to the total variance of the data set. For visualization, we projected the top two principal components, times the square roots of their corresponding eigenvalues (since the eigenvalues are variances in Å^2^) multiplied by an array of unitless scalars between −5 and +5 (to facilitate easier observation), onto the corresponding representative structure, saving modified structures and compiling them into a movie. The representative structure for each system was defined by the simulation snapshot with the lowest RMSD with respect to the geometric average of the entire analyzed trajectory. Lastly, we projected the CENP-A and H3 trajectories onto the first two principal components, calculated by C*α* PCA, to reveal the conformational space explored by each system studied.

## Additional Information

**How to cite this article**: Winogradoff, D. *et al.* Shearing of the CENP-A dimerization interface mediates plasticity in the octameric centromeric nucleosome. *Sci. Rep.* **5**, 17038; doi: 10.1038/srep17038 (2015).

## Supplementary Material

Supplementary Information

Supplementary Movie 1

Supplementary Movie 2
