# Supplementary material for: Shearing of the CENP-A dimerization interface mediates plasticity in the octameric centromeric nucleosome
Source: Sci Rep. 2015 Nov 25;5:17038. doi: 10.1038/srep17038 (PMC4658507; doi:10.1038/srep17038)
Supplement: Supplementary Information [file srep17038-s1.pdf]

Supplementary Information: Shearing of the CENP-A dimerization interface mediates plasticity in the octameric centromeric nucleosome.

David Winogradoff[1], Haiqing Zhao[2,3], Yamini Dalal[3,*], and Garegin A. Papoian[1,2,4,*]

[1]Chemical Physics Program, University of Maryland, College Park, MD 20742, USA
[2]Biophysics Program, University of Maryland, College Park, MD 20742, USA
[3]Laboratory of Receptor Biology and Gene Expression, National Cancer Institute, National Institutes of Health, Bethesda, MD, USA
[4]Department of Chemistry and Biochemistry, University of Maryland, College Park, MD 20742, USA
[*]G.A.P. (email: gpapoian@umd.edu) or Y.D. (email: dalaly@mail.nih.gov).

Glossary of terms

1. **RMSD** Root-mean-square deviation is a measure of difference between two structures after alignment:

$$\sqrt{\frac{1}{N}\sum_{i=1}^{N}\delta_i^2}, \tag{1}$$

where δ is the distance between N pairs of equivalent atoms (Cα atoms, for example).

2. **RMSF** Root-mean-square fluctuation is a measure of the deviation, over time, between a particle (i) and some reference position:

$$\sqrt{\frac{1}{T}\sum_{j=1}^{T}(x_i(t_j)-\langle x_i\rangle)^2}, \tag{2}$$

where T is the total time (i.e. total number of simulation snapshots considered) and $\langle x_i\rangle$ is the time-averaged position of that particle, serving as a reference point.

3. **COM** Center-of-mass is the geometric average location of a distribution of mass in space. For a system of particles P_i, i = 1, 2, ..., n, with masses m_i, summing to M, located in space with coordinates r_i,

$$COM = \frac{1}{M}\sum_{i=1}^{n} m_i r_i. \tag{3}$$

For our purposes, proteins are represented by their corresponding Cα atoms.

4. **PCA** Principal Component Analysis is a statistical method that transforms a set of observations (Cα positions in our case) of possibly correlated variables into a set of orthogonal, uncorrelated variables known as principal components. The first principal component accounts for the greatest possible variance, the second principal component accounts for the second-most, and so-forth.

5. **Pseudo-dyad** (or dyad axis) of the nucleosome core particle is an axis of symmetry perpendicular to the superhelical axis, dividing the nucleosomal DNA in two and bisecting the histone homotetramer through its central four-helix bundle.

6. **Acidic patch** is a cluster of eight acidic residues (H2A E56, E61, E64, D90, E91, E92 and H2B E102, E110) that forms a pocket at the surface of the nucleosome.

7. **Histones** are positively charged proteins found in the nuclei of eukaryotic cells, which order and package DNA into nucleosomes, the fundamental structural units of the protein-DNA complex known as chromatin. Within the nucleosome core particle, there are four types of core histones (H2A, H2B, H3, H4), each of which have histone variants except H4.

8. **CENP-A** CENtromere Protein-A is a histone variant of H3 found at the centromere, defining the location to which microtubules bind during mitosis.

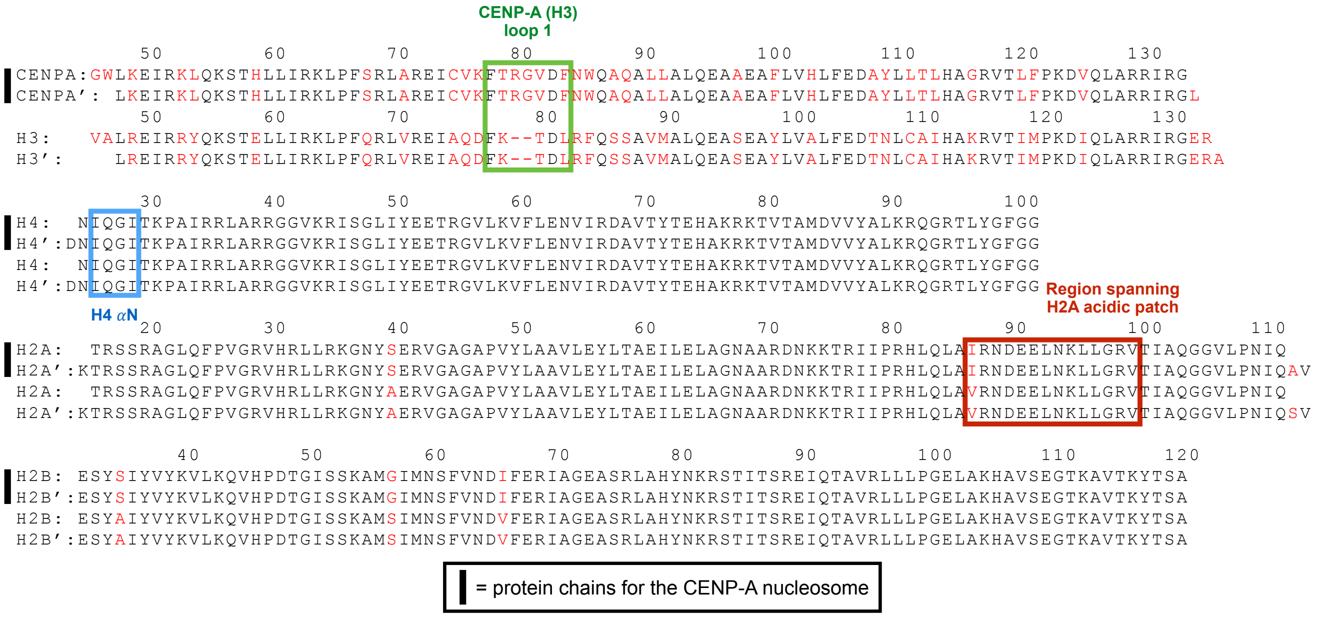

Figure 1. Amino acid sequences of the H3 and CENP-A systems. Amino acid sequence alignment for the H3 and CENP-A systems. Boxes highlight important regions.

Table 1. Distances between dimers within H3 and CENP-A tetramers (Å)

System	H3/H4 to H3'/H4'	H3/H4 to H2A/H2B	H3'/H4' to H2A'/H2B'
H3 nucleosome	34.3 $\pm$ 0.18	33.4 $\pm$ 0.27	32.4 $\pm$ 0.21
CENP-A nucleosome	34.7 $\pm$ 0.22	33.7 $\pm$ 0.34	32.8 $\pm$ 0.30
H3 octamer	33.9 $\pm$ 0.21	33.7 $\pm$ 0.25	32.8 $\pm$ 0.27
CENP-A octamer	34.5 $\pm$ 0.29	33.6 $\pm$ 0.39	33.0 $\pm$ 0.35

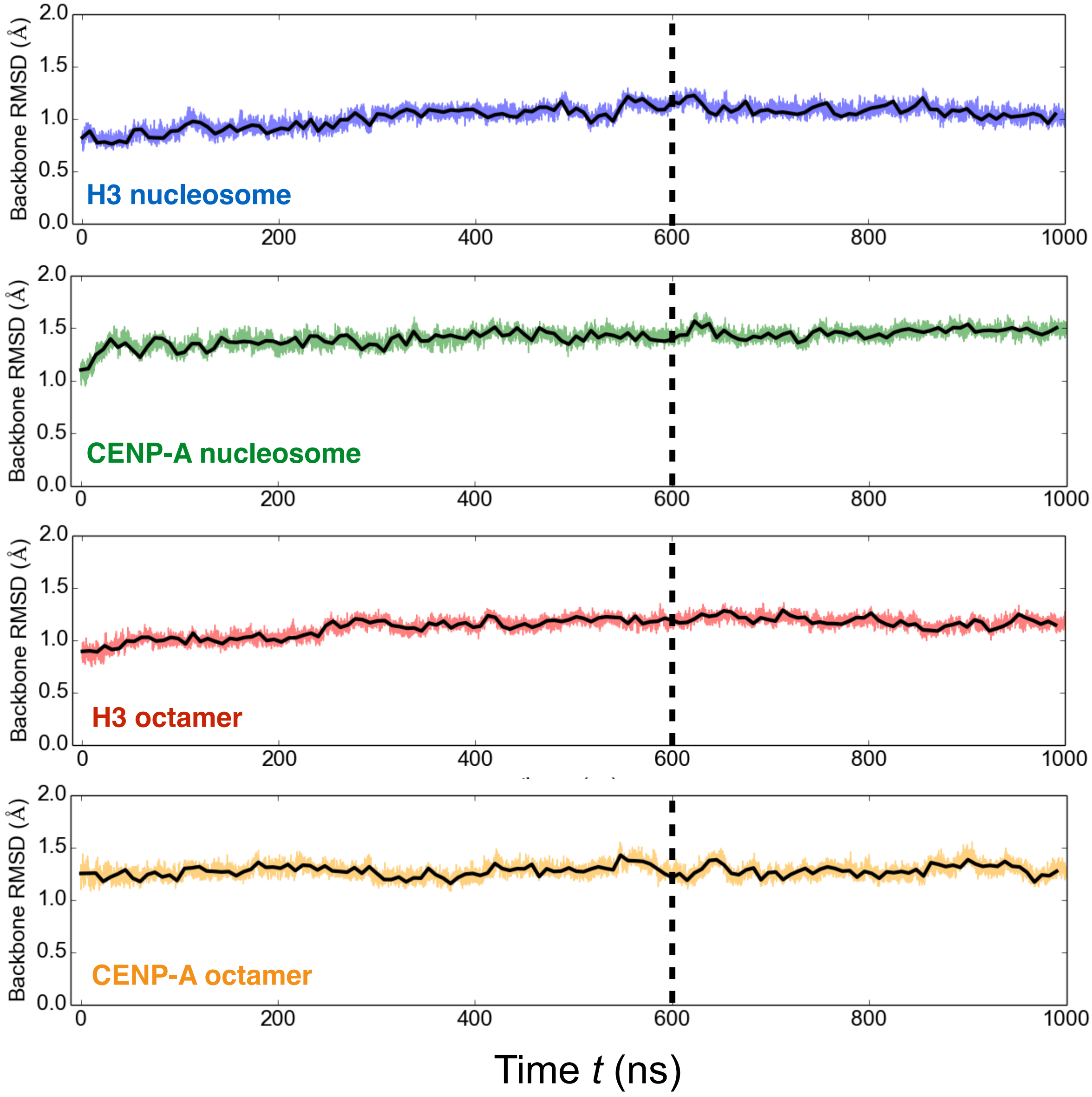

Figure 2. H3 and CENP-A systems reach stable equilibrium after 600 ns. Protein backbone RMSD to the energy-minimized structures as a function of time for the H3 and CENP-A systems. The solid black lines represent running averages. The vertical dashed lines indicate the amount of time removed to ensure a stable equilibrium for each system.

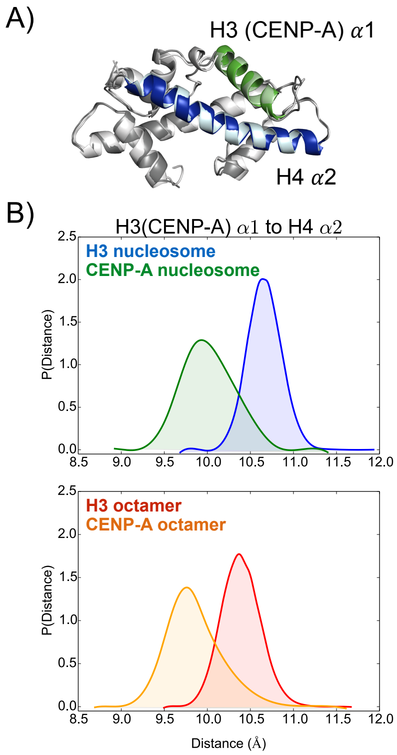

Figure 3. CENP-A/H4 is more compact, on average, than H3/H4 in the context of the nucleosome and octamer structures. (A) Crystal structure distances between H3 α1 and H4 α2 (9.9 Å), and between CENP-A α1 and H4 α2 (10.1 Å). (B) Probability density functions for the distances between these two specific helices for the H3 and CENP-A systems. Structure figures drawn in Pymol.

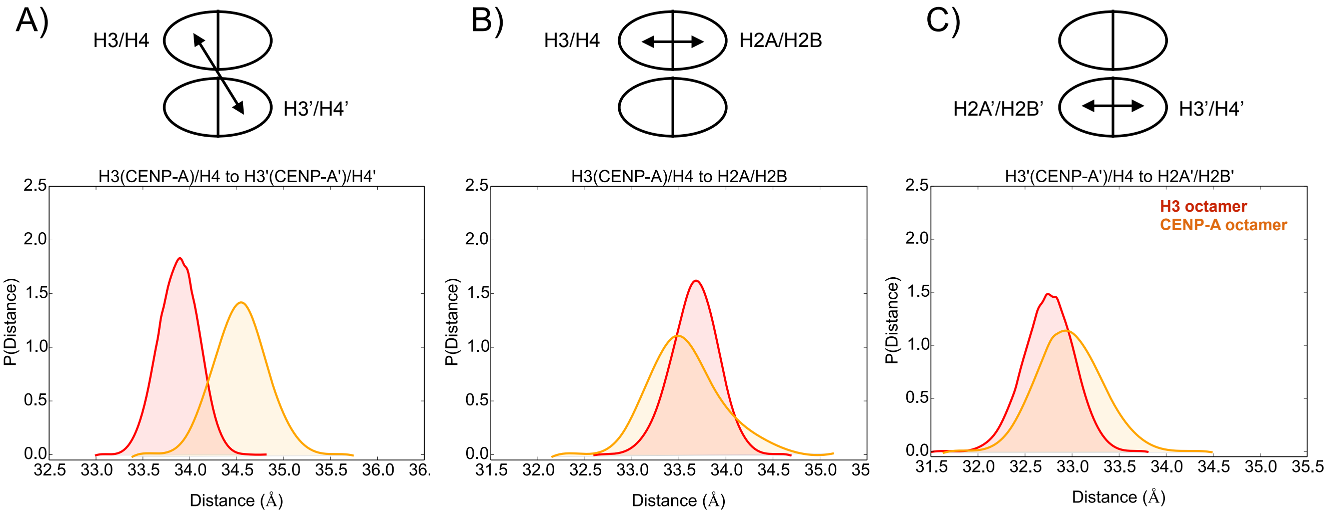

Figure 4. The CENP-A octamer exhibits greater global flexibility than the H3 octamer Probability density functions for the distances within tetramers for the H3 and CENP-A octamers.

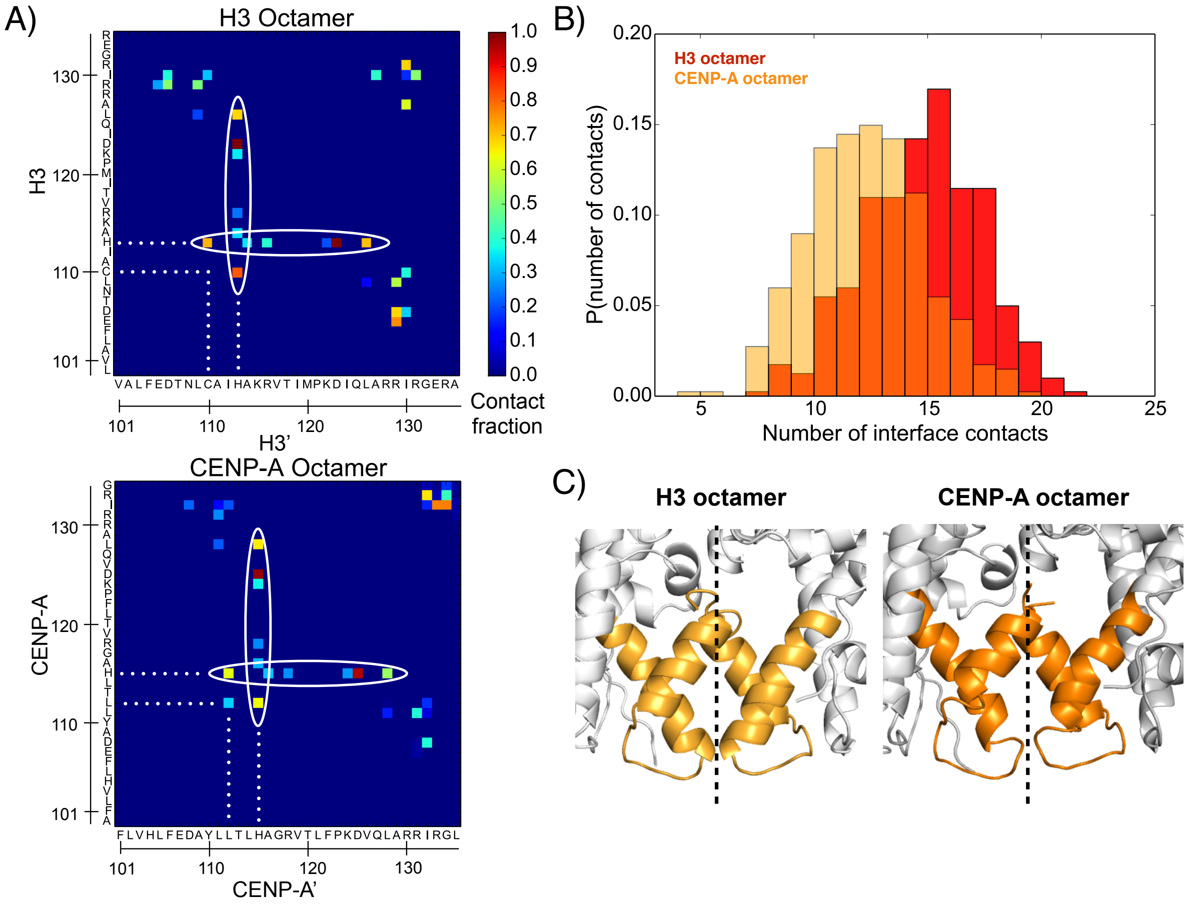

Figure 5. The CENP-A octamer dimerization interface is formed by fewer, and weaker, contacts than the corresponding interface in the H3 octamer. (A) Contact maps for the dimerization interfaces of the H3 and CENP-A octamers. (B) Histograms of the number of H3–H3' and CENP-A–CENP-A' contacts. (C) The dimerization interfaces for H3–H3' (light orange) and CENP-A–CENP-A' (dark orange). The dashed line represents the pseudo-dyad. Structure figures drawn in Pymol.

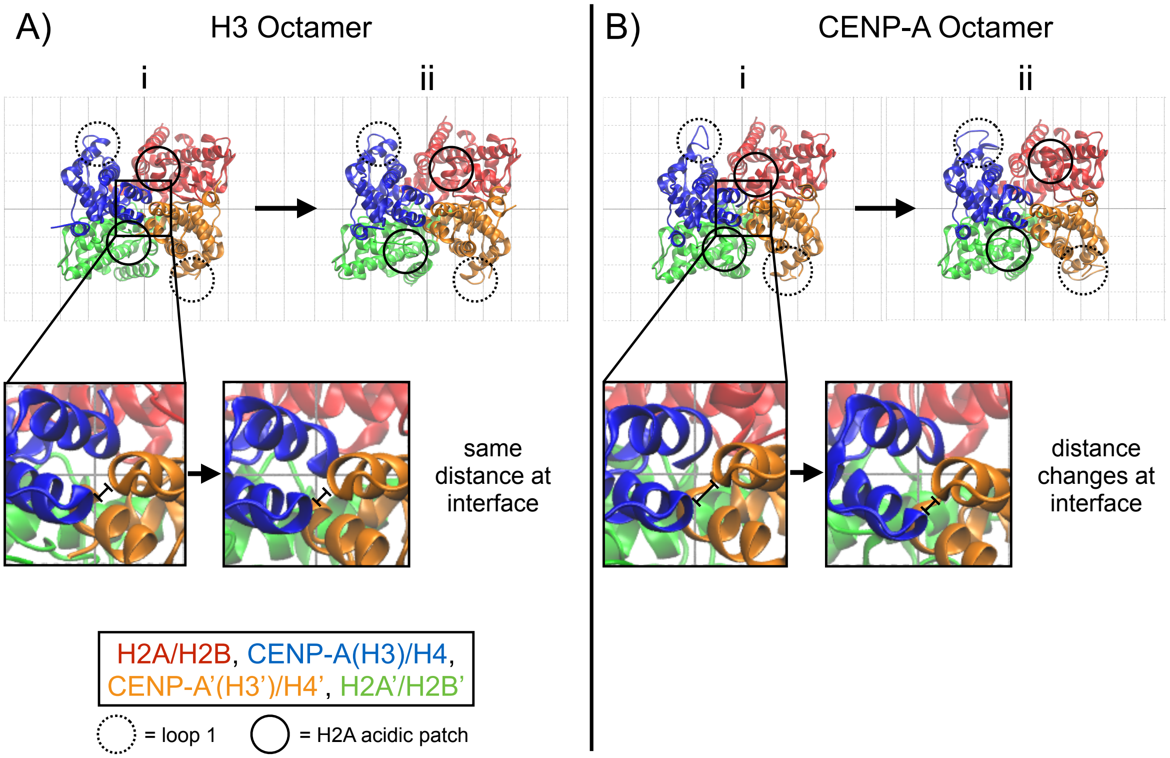

Figure 6. Greater distortion at the CENP-A dimerization interface, relative to the H3 octamer, is a major mode of motion of the CENP-A octamer. (A) Two-dimensional plots displaying the top principal component for the H3 octamer viewed from the side of the DNA supercoil axis. (B) Plots for the top principal component of the CENP-A octamer. The insets display the motion of the H3–H3' and CENP-A–CENP-A' dimerization interfaces. Structure figures drawn in VMD.

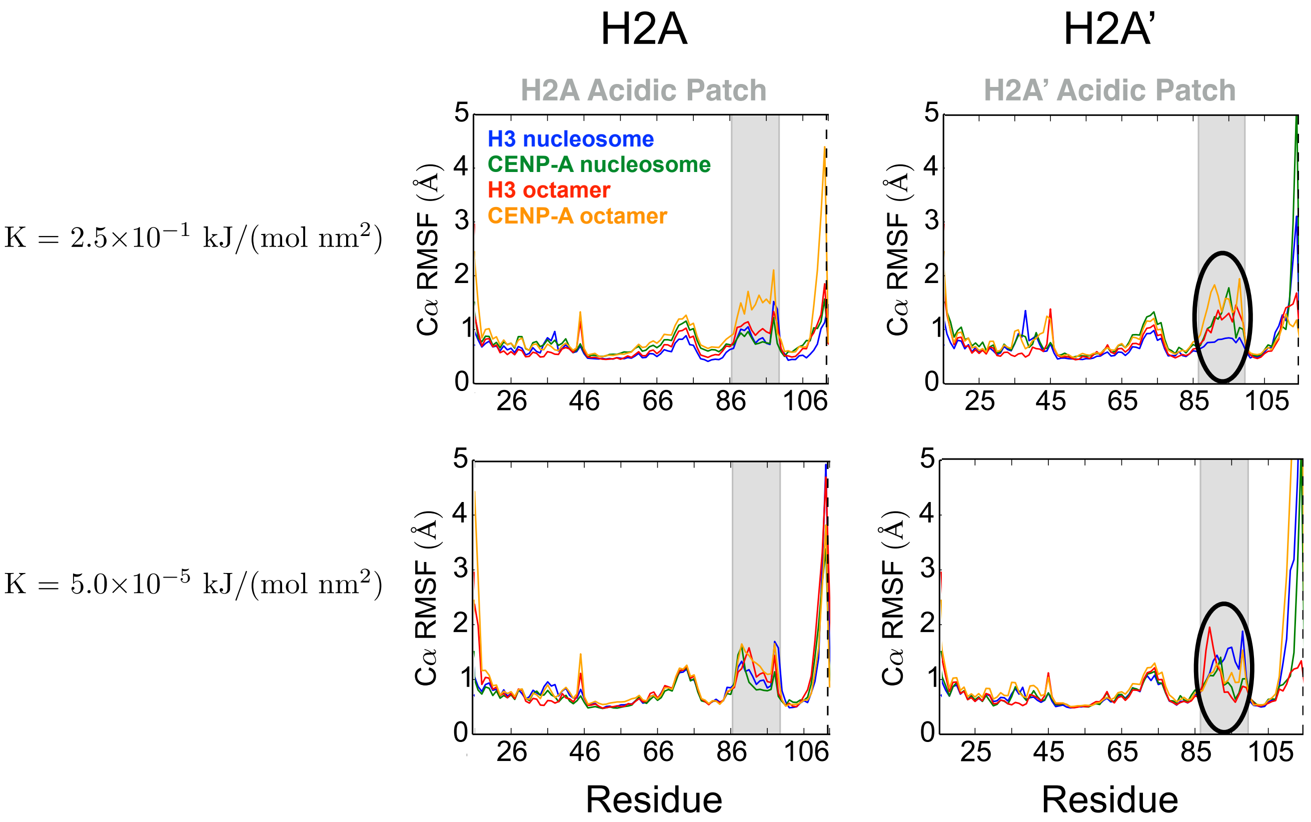

Figure 7. **Asymmetric variability in H2A acidic patch mobility.** Cα RMSF of proteins H2A and H2A' in all four systems considered. Gray boxes define the regions spanning the H2A acidic patch (residues 87-100) in both of the heterotypic halves of the protein cores. Labels along the top identify the heterotypic half, and labels along the left-hand side indicate the strength of harmonic position restraints applied to heavy atoms. Black ovals highlight differences in H2A' acidic patch mobility, discussed further in the main text.

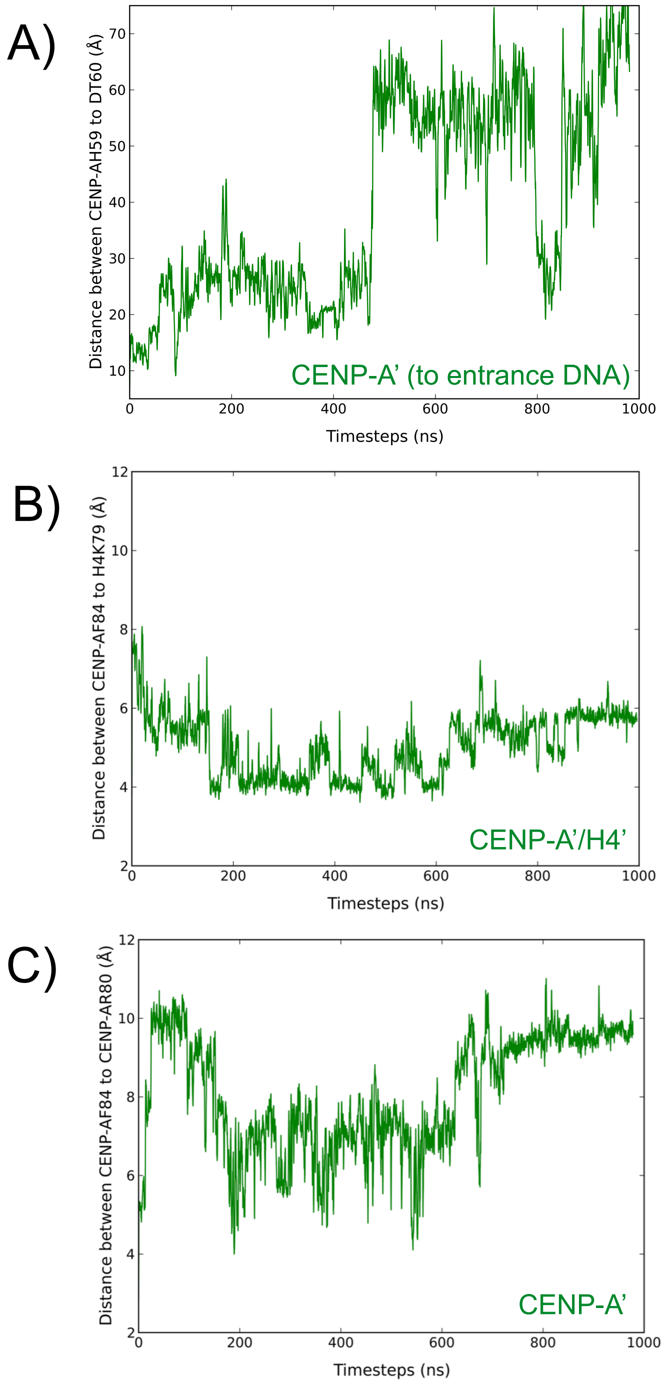

Figure 8. Key residue substitutions in CENP-A contribute to DNA instability near the pseudo-dyad. As a function of time, we traced the distances between (A) CENP-A' His 59 and bp -60, (B) CENP-A' F84 and H4' K79, and between (C) CENP-A' F84 and CENP-A' R80. We specifically measured (A) NE2 of His 59 to P atom of DA-60; (B) CE1 of F84 to CB atom of K79; and (C) CE1 of F84 to CB atom of R80. CENP-A' His 59 interacts with entry DNA until detachment occurs, and interactions between CENP-A' R80, F84 and H4' K79 develop a hydrophobic pocket, weakening the CENP-A protein core affinity for DNA.

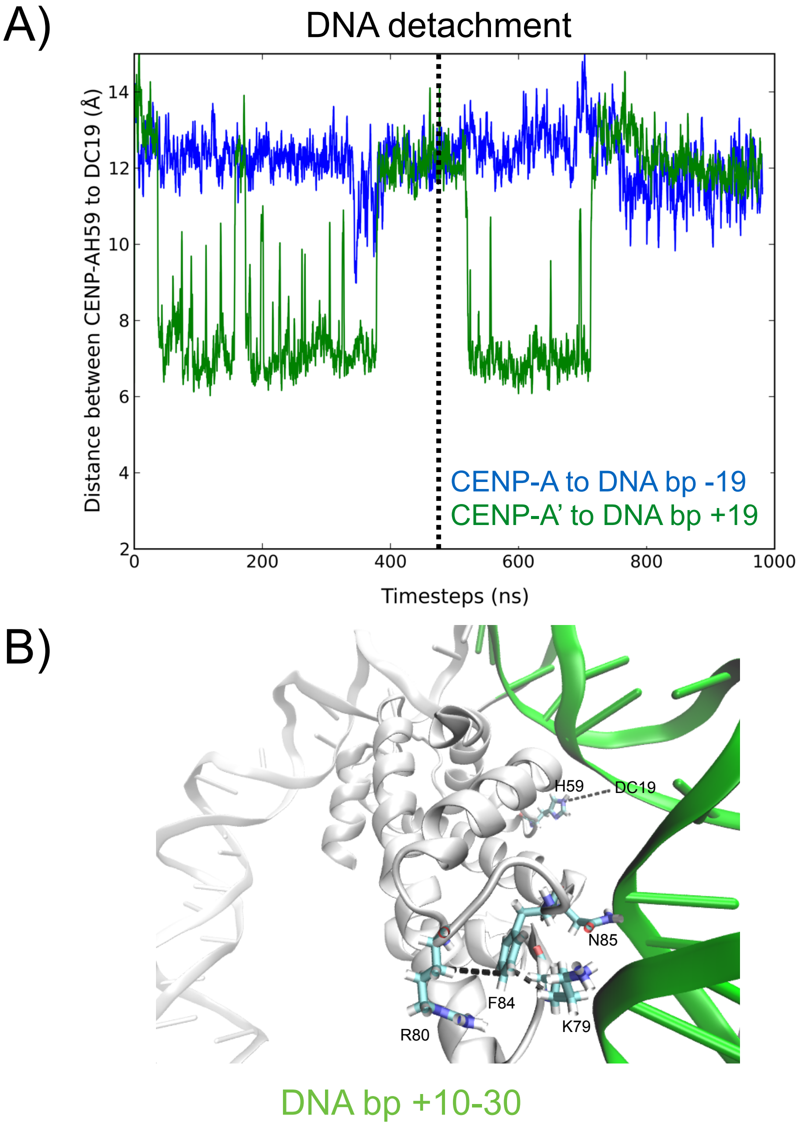

Figure 9. The asymmetric role of CENP-A H59 in DNA flexibility. (A) Time traces of the distances between CENP-A H59 and DNA bp -19, and between CENP-A' H'59 and DNA bp +19. This illustrates that CENP-A' H'59 and CENP-A H59 play different roles in pseudo-dyad proximal DNA structural flexibility. (B) Residues from the CENP-A nucleosome, shown as sticks, that contribute to the relative instability of CENP-A nucleosomal DNA from basepairs +10 to +30. Structure figure drawn in VMD.

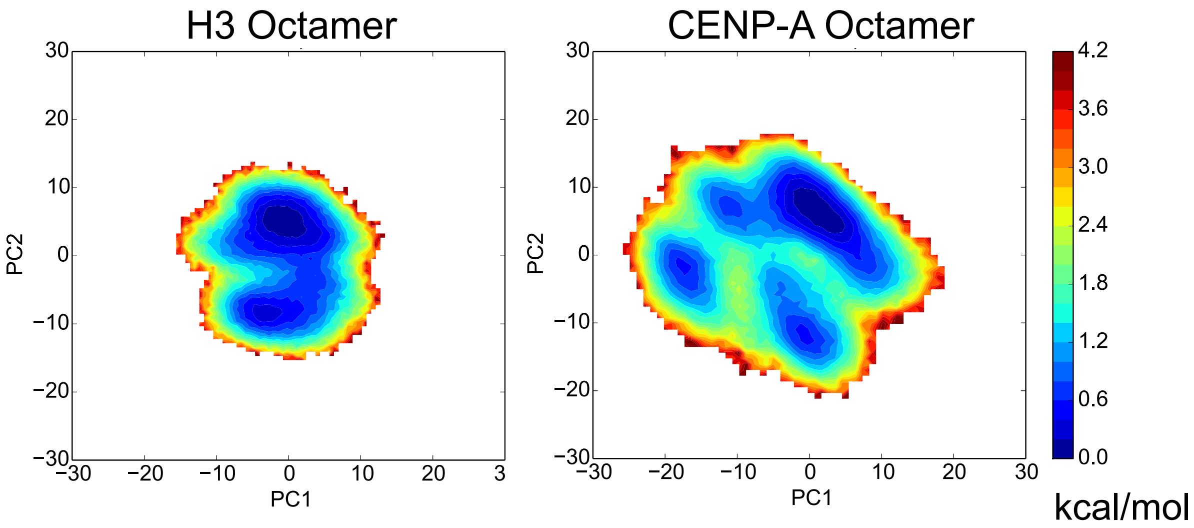

Figure 10. The characteristic free energy landscape is more rugged for the CENP-A octamer than for the H3 octamer. Free energy projection of the H3 and CENP-A octamers onto their respective first two principal components.

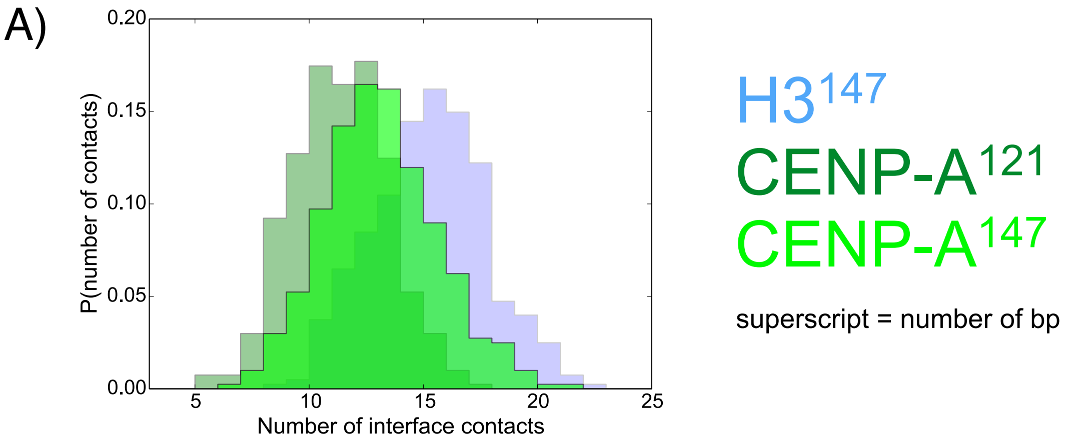

B)

Distances between dimers (Å)

System	H3/H4 to H3'/H4'	H3/H4 to H2A/H2B	H3'/H4' to H2A'/H2B'
$H3^{147}$	34.3 ± 0.18	33.4 ± 0.27	32.4 ± 0.21
CENP-A^{121}	34.7 ± 0.22	33.7 ± 0.34	32.8 ± 0.30
CENP-A^{147}	34.7 ± 0.24	32.8 ± 0.25	32.8 ± 0.27

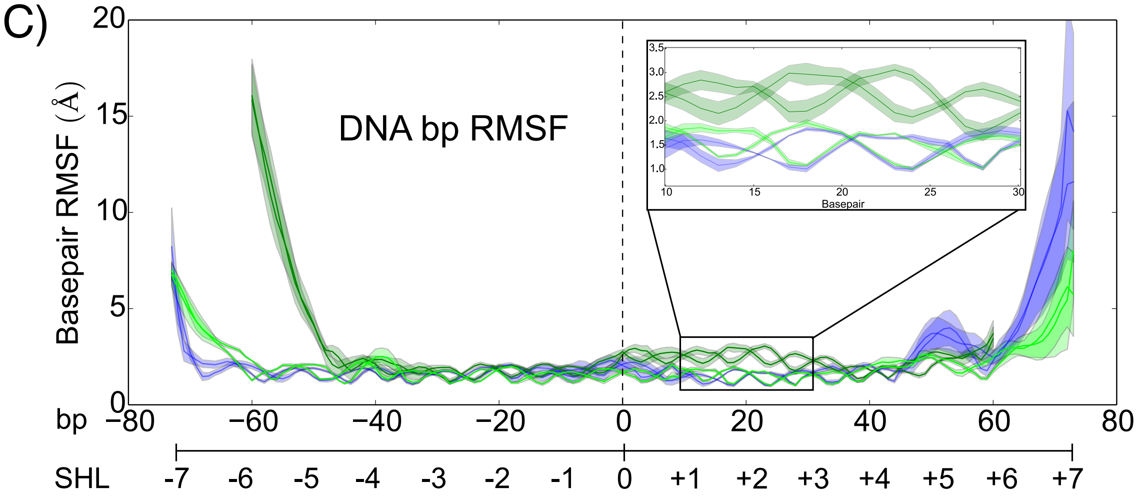

Figure 11. The effects of DNA length and sequence on CENP-A nucleosome dynamics. A comparison between the CENP-A nucleosome with the 121 bp from 3AN2, denoted CENP-A^{121}, and with the 147 bp from 1KX5, denoted CENP-A^{147}, to the canonical H3 nucleosome, denoted $H3^{147}$. (A) Histograms of the number of CENP-A:CENP-A' dimerization interface contacts. (B) Distances between histone dimers, the red box highlighting the most significance difference. (C) Root mean squared fluctuations (RMSF) per base pair. The shaded areas represent $\pm$ one standard deviation. Two lines for each system correspond to the two strands of DNA. With the longer sequence from the crystal structure for the canonical H3 nucleosome (PDB ID 1KX5), the CENP-A nucleosomal DNA base pairs +10 to +30 remain more stably associated with the histone core.

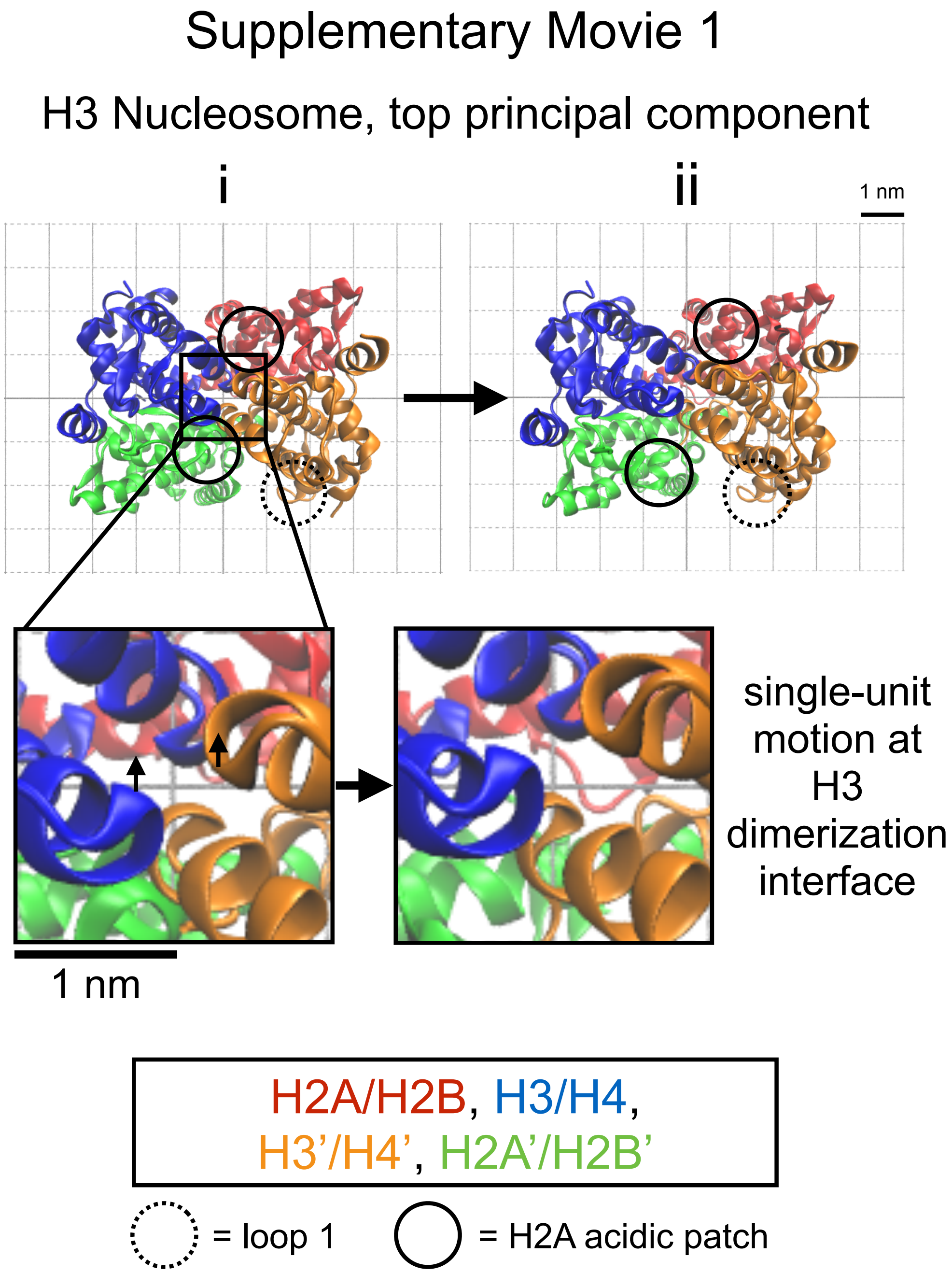

Figure 12. **The top principal component of the canonical H3 nucleosome.** Two-dimensional plot displaying the top principal component for the canonical H3 nucleosome viewed from the side of the DNA supercoil axis. The insets display the motion of the H3 dimerization interface. Structure figures drawn in VMD.

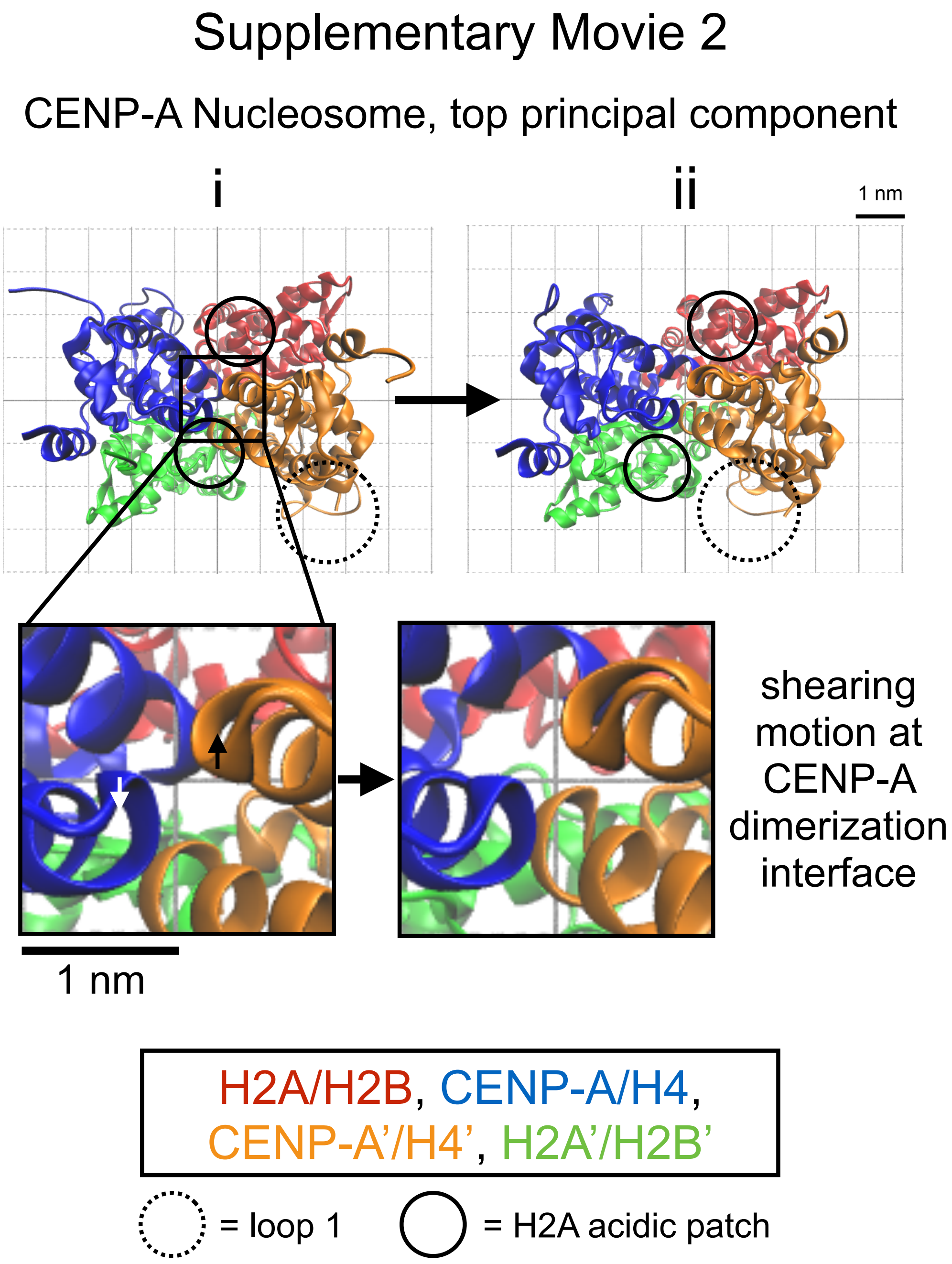

Figure 13. The top principal component of the CENP-A nucleosome. Two-dimensional plot displaying the top principal component for the CENP-A nucleosome viewed from the side of the DNA supercoil axis. The insets display the motion of the CENP-A dimerization interface. Structure figures drawn in VMD.
